# Characterization of the Miiuy Croaker (*Miichthys miiuy*) Transcriptome and Development of Immune-Relevant Genes and Molecular Markers

**DOI:** 10.1371/journal.pone.0094046

**Published:** 2014-04-08

**Authors:** Rongbo Che, Yueyan Sun, Dianqiao Sun, Tianjun Xu

**Affiliations:** Laboratory of Fish Biogenetics & Immune Evolution, College of Marine Science, Zhejiang Ocean University, Zhoushan, China; University of Lausanne, Switzerland

## Abstract

**Background:**

The miiuy croaker (*Miichthys miiuy*) is an important species of marine fish that supports capture fisheries and aquaculture. At present commercial scale aquaculture of this species is limited due to diseases caused by pathogens and parasites which restrict production and limit commercial value. The lack of transcriptomic and genomic information for the miiuy croaker limits the ability of researchers to study the pathogenesis and immune system of this species. In this study we constructed a cDNA library from liver, spleen and kidney which was sequenced using Illumina paired-end sequencing to enable gene discovery and molecular marker development.

**Principal Findings:**

In our study, a total of 69,071 unigenes with an average length of 572 bp were obtained. Of these, 45,676 (66.13%) were successfully annotated in public databases. The unigenes were also annotated with Gene Ontology, Clusters of Orthologous Groups and KEGG pathways. Additionally, 498 immune-relevant genes were identified and classified. Furthermore, 14,885 putative simple sequence repeats (cSSRs) and 8,510 putative single nucleotide polymorphisms (SNPs) were identified from the 69,071 unigenes.

**Conclusion:**

The miiuy croaker (*Miichthys miiuy*) transcriptome data provides a large resource to identify new genes involved in many processes including those involved in the response to pathogens and diseases. Furthermore, the thousands of potential cSSR and SNP markers found in this study are important resources with respect to future development of molecular marker assisted breeding programs for the miiuy croaker.

## Introduction

Miiuy croaker (*Miichthys miiuy*), which belongs to family Sciaenidae, is a commercially important marine fish found in Northwest Pacific: from western Japan to the East China Sea [1]. This species supports important commercial fisheries and aquaculture activities within this area. In China, the miiuy croaker, due to its fast growth, various feeding habit, medicinal value and high economic value is an important aquaculture species that has been widely cultured since late 1990s [2].

Previous studies on miiuy croaker have paid more attention to understand its life habits for establishing appropriate technology on artificial propagation, especially breeding high quantity fish fry [3], [4]. Over the last decade, many diseases caused by pathogens and parasites have had significant effects on aquaculture production of this species and the economic value of the industry [5], [6]. To improve the economics of miiuy croaker culture new procedures and tools including selective breeding programs for disease resistance need to be developed to reduce the impacts of disease. Prerequisite conditions to the development of such procedures, tools and programs is knowledge of the genes and pathways involved in infection and disease and information to allow for molecular marker development [7], [8]. Prior to this study transcriptomic and genomic information for the miiuy croaker was limited to studies on immune-related genes. These studies include chemokines [9]–[11], C9 [12], MHC I, II [13], [14], Hepcidin [15] and cathepsins [16], [17], and a suite of 193 putative immune-related genes identified by EST sequencing [18]. With respect to molecular markers only a few markers developed from genomic DNA and ESTs in miiuy croaker have been reported [19], [20]. Taken together these data are far from sufficient to support the development of a linkage map or markers which are necessary to support the development of marker assisted selection breeding programs for traits on interest in this species. A fast and cost-efficient approach to exploit molecular markers for miiuy croaker is required.

Over the last decade, a large number of transcriptomic and genomic sequences became available in model organisms [21]–[26], which have greatly improved the understanding of complexity molecular mechanism, especially developing large-scale genetic basis knowledge. Recent advances in high throughout sequencing technologies, including the Roche/454 Genome Sequencer FLX Instrument, the ABI SOLiD System, and the Illumina Genome Analyser, have advanced research in many fields, especially *de novo* transcriptome sequencing for non-model organisms [27]–[30]. These technologies have been used to obtain genomic and transcriptomic information, which includes the identification of immune-related genes, in species of marine fishes such as Japanese sea bass (*Lateolabrax japonicas*) [31], Atlantic salmon (*Salmo salar*) [32], Asian seabass (*Lates calcarifer*) [33] and rainbow trout (*Oncorhynchus mykiss*) [34]. In addition, large-scale simple sequence repeat (SSR) markers or single nucleotide polymorphisms (SNP) markers were developed based on transcriptome sequencing [35]–[38]. Compared with the other two platforms of next generation sequencing technology, the Illumina Genome Analyser is more efficient and inexpensive and can produce more sequences with greater coverage using the recent algorithmic [39] and experimental technology. Greater coverage allows for the identification of rare genes and supports the assembly of transcripts [40].

In the present study, a non-normalized cDNA library was generated from three immune tissues (liver, spleen and kidney) and sequenced using Illumina paired-end sequencing technology. The sequence data was used to characterize the transcriptome with an emphasis on immune-related genes as well as to identify potential molecular cSSR and SNP markers. We also isolated and validated a set of cSSR markers and assessed the polymorphism of these mined loci. As far as we know, this is the first comprehensive report on the transcriptome of miiuy croaker. The transcriptome data generated from our study are useful for gene annotation and discovery, developing molecular markers and assembling genomic and transcriptomic in miiuy croaker. Furthermore, the markers developed and validated in this study have increased the number of molecular markers available for this species and form an important resource for future mapping and marker assisted selection breeding program efforts for the miiuy croaker.

## Results and Discussion

### Illumina Paired-end Sequencing and *De novo* Assembly

**Figure 1:**
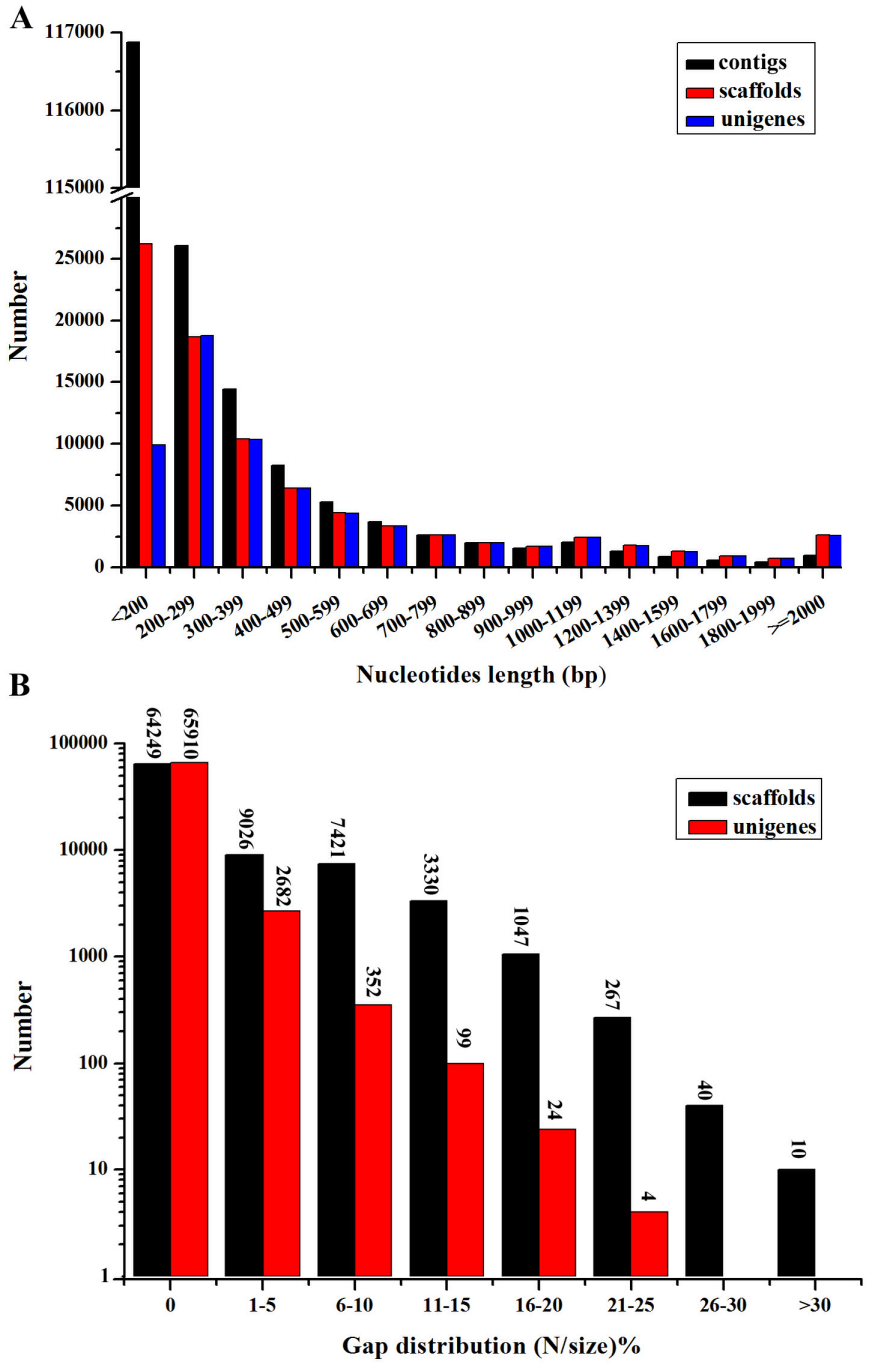
Analysis of Illumina short read assembly quality. (**A**) Size distributions of de novo assembled contigs, scaffolds, and unigenes. (**B**) Gap distributions of assembled scaffolds and unigenes.

To characterize the transcriptome of the miiuy croaker (*Miichthys miiuy*), with an emphasis on immune-related genes, a non-normalized cDNA library was generated using equal amounts of RNA extracted from tissues with known immune function (liver, spleen and kidney). This library was sequenced using Illumina paired-end sequencing and after strict data cleaning and quality testing 25,760,602 high-quality reads were obtained with 95.29% Q20 bases (Table 1). These remaining high-quality reads were assembled using the short reads assembly program SOAPdenovo [41]. According to the overlapping information of high-quality reads, a total of 186,917 contigs were generated with an average length of 275 bp and a N50 of 412 bp (Table 1). The length of contigs ranged from 75 to 6,934 bp, with 37.46% of the contigs having a length of more than 200 bp (Fig. 1A). SOAPdenovo allowed us to map the reads back to contigs, with the help of paired-end reads, it is possible to identify contigs derived from the same transcript as well as the distances between these contigs. Then, we joined these contigs into scaffolds using “N” to represent unknown nucleotides between each two contigs inferred from the paired-end information. As a result, 85,389 scaffolds were obtained. The length distribution of scaffolds was given in Fig. 1A. Of these, 64,249 scaffolds (approximately 75.2%) did not contain gap regions, whereas the gap region lengths of 16,477 scaffolds (about 19.3%) were less than 10% of their corresponding scaffolds (Fig. 1B). To further shorten the remaining gaps, paired-end reads were used to fill scaffold gaps. We gathered the paired-end reads with one end mapped on the unique contig and the other located in the gap region, and filled the small gaps within the scaffolds. The resulting sequences without redundancy, containing the least Ns and not being extended on either end, were defined as unigenes. The subsequent analyses were all based on the obtained unigenes. With the steps mentioned above, more than half of the gaps were filled and 69,071 unigenes were finally obtained in this research with only approximately 0.06 Mb of gaps (0.14% of the total unigene sequences) remained unclosed and an average length of 572 bp and N50 (median unigene) length of 826 bp (Table 1). The length of assembled unigenes ranged from 150 to 9,883 bp, and 23,634 unigenes (34.22%) had the length over 500 bp (Fig. 1A). Among the assembled unigenes, 65,910 unigenes (about 95.42%) did not contain gap region, whereas only 3,161 unigenes (approximately 4.58%) were padded with Ns. The gap length distribution within the assembled unigenes was shown in Fig. 1B.

**Table 1:** Summary of the miiuy croaker transcriptome.

Description	Number
High-quality reads	25,760,602
Total Nucleotides of high-quality reads (bp)	2,318,454,180
Q20 percentage	95.29%
GC percentage of high-quality reads	49.86%
Number of contigs	186,917
Range of contigs length (bp)	75–6,934
Average and N50 length of contigs (bp)	275/412
Number of unigenes	69,071
Range of unigenes length (bp)	150–9,883
Average and N50 length of unigenes (bp)	572/826

### Evaluation and Validation of Assembled Transcripts

**Figure 2:**
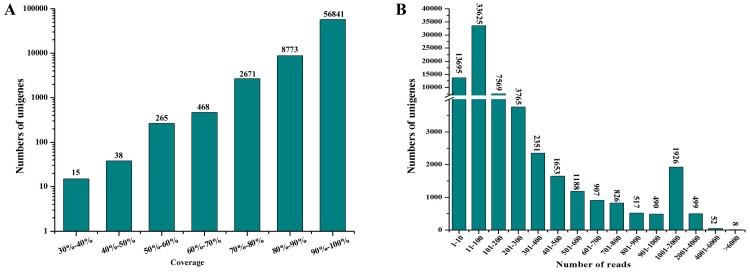
Evaluation and Validation of assembled transcripts. (**A**) Coverage distribution of assembled unigenes. (**B**) Distribution of unique mapped reads of the assembled unigenes.

To evaluate the quality and coverage of the assembled unigenes the short reads alignment tool, SOAPaligner [42] was used to realign all usable sequence reads with the unigenes. In this report, the sequencing depth of the assembled unigenes ranged from 0.3 to 2,168 folds, with an average of 23.95 folds. The coverage of the assembled unigenes ranged from 30.30% to 100.00% and the higher coverage depth had the more number of assembled unigenes (Fig. 2A). It is important to note that, in some degree, increased coverage depth can result in higher coverage of the coding regions [43]. The distribution of RPKM (Reads per kb per million reads) values which can estimate the unigenes expression level indicated that most unigenes were expressed at high levels. Among 69,071 unigenes, 95.00% (about 65,617) had RPKM values of more than 10 and 58.00% (about 40,061) had RPKM values of no less than 100. In the number of the unigenes, a total of 55,376 (about 80.17%) were remapped by more than 10 reads, 21,751 (almost 31.49%) were remapped by more than 100 reads, and 2,485 (approximately 3.60%) were remapped by more than 1000 reads, while only 8 unigenes were remapped by more than 8000 reads (Fig. 2B). Taken together all of these data indicated that there was good coverage of the assembled unigenes by the sequencing reads.

**Figure 3:**
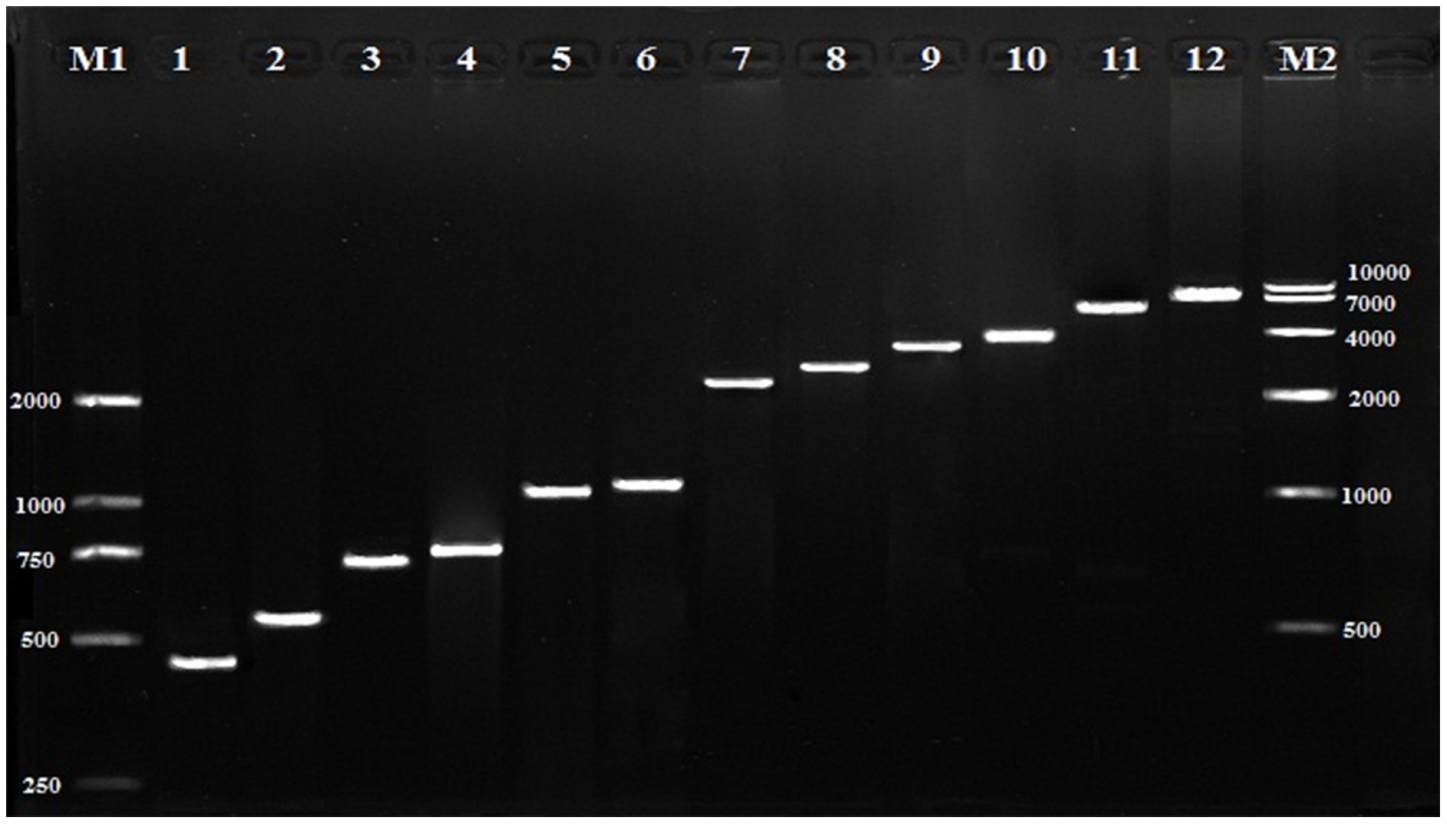
RT-PCR amplification and agarose gel (1%) electrophoresis of twelve transcripts. The corresponding run lanes of Unigene 5, Unigene 62288, Unigene 10257, Unigene 68579, Unigene 10014, Unigene 68682, Unigene 20918, Unigene 69000, Unigene 10657, Unigene 4806, Unigene 20628 and Unigene 21126 are from 1 to 12, respectively. And the amplification lengths of these unigenes were given in table 2.

To validate the assembled transcripts, twelve unigenes were selected for RT-PCR (reverse transcription polymerase chain reaction) amplification. Their putative gene names, primer sequences and expected PCR product sizes are shown in Table 2. As we expected, all 12 unigenes gave amplicons of expected sizes (Fig. 3). The results of these two methods for evaluation and validation of assembled transcripts not only testified the accuracy of Illumina paired-end sequencing and *de novo* assembly, but also indicated that our study could be useful for further research.

**Table 2:** Putative gene name, primer sequences and the expected size for RT-PCR of the twelve unigenes.

Number	Putative Gene Name	Forward primer	Reverse primer	Product size (bp)
				
Unigene5	Cell adhesion molecule 4	ATGGGAGCTTCCAGGTGTAT	ATGTTTGCGGGTCATCAGTA	406
Unigene62288	Myeloperoxidase	GCCTTTCGGTTGGGAGACAC	CCTCTTTGTGCTGGGCGTCT	535
Unigene10257	Melanoma-associated antigen G1(MAGE)	TCTGAGGGTCCTGTTCGTGG	GCAGTCCGGCAACTTAGTGT	680
Unigene68579	Glycogen [starch] synthase, muscle	TCATAATCCATCGGAAGTAGAGG	TAAGGAAGCAGGCGACAGAC	792
Unigene10014	Diphosphomevalonate decarboxylase	GCAACCCAGTAGATCCAAGT	CAAAGAGGAGGACATAACCC	980
Unigene68682	Copper transporting ATPase 1	CAAGATGCTACGACGTGTCC	TGGCGATATTGTCCTGAATG	1,070
Unigene20918	E3 ubiquitin-protein ligase UBR5	GGAAATGGCACAATCTACCC	CCTCACCTGCTACTCCCTCTAC	2,311
Unigene69000	Na+/K+ ATPase alpha subunit isoform 6	TCCTCACTAACAGGCGAGTC	GGGCGGGTCAATGTAGAA	2,625
Unigene10657	Cytoplasmic dynein 1 heavy chain	ACAAGCCCATATCAACCC	GCAAACGCTGTCCATTAC	3,273
Unigene4806	Ankyrin repeat and KH domain-containing protein 1	CTAAGCGTGAGAAGCGTAAGG	AAGAATGGCGTCAAGACAACT	3,715
Unigene20628	Plectin a	AAAAGCAGGCTGACGATGAG	TCTGTGGGAAGGCGGAAGTA	5,005
Unigene21126	E3 ubiquitin-protein ligase HUWE1 isoform 2	CGGCTAAGGTAAAGGCAAGT	CAGGAGTAGGCAAATCTAAATCA	7,318

### Characterization of the Nonredundant Unigenes of Miiuy Croaker by Searching against Public Databases

**Figure 4:**
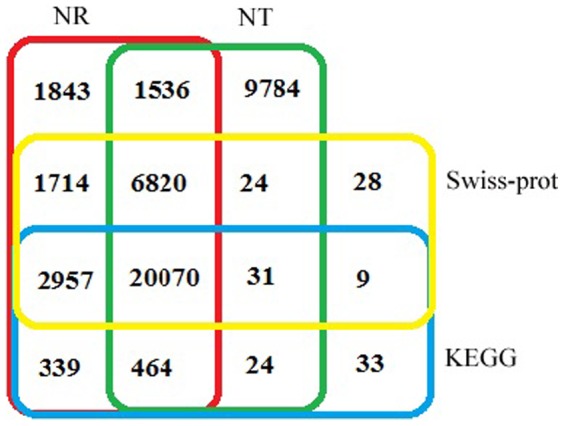
Comparison of the number of unigene annotations obtained from the different databases. The number of unigene annotations hits from the NR, NT, Swiss-Prot and KEGG databases (E-value ≤1.0E-5), respectively.

**Figure 5:**
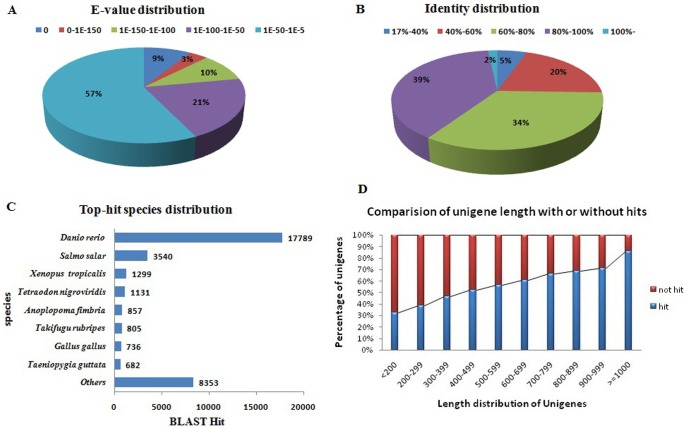
Characteristics of homology search of assembled unigenes against NR databases. (**A**) E-value distribution of BLAST hits for each unigene with a cutoff E-value of 1.0E-5. (**B**) Identity distribution of the top BLAST hits for each unigene. (**C**) BLASTx top-hit species distribution of gene annotations against NR databases. (**D**) Length of unigenes with hits compared with those without hits.

For better validation and annotation of the all nonredundant unigenes, 69,071 unigenes were searched against public protein and nucleotide databases of the National Center for Biotechnology Information (NCBI) using BLASTX and BLASTN algorithm with an E-value threshold of 1.0E-5. As a result, 38,753 (56.11%), 35,743 (51.75%), 31,653 (45.83%) and 23,927 (34.64%) of the 69,071 miiuy croaker unigenes had significant matches with sequences in NT (Nonredundant nucleotide), NR (Nonredundant protein), Swiss-Prot and KEGG (Kyoto Encyclopedia of Genes and Genomes) databases, respectively. Among the unigenes, 31,561 (45.69%) were synchronously annotated by NR and Swiss-Prot, 23,027 (33.34%) by NR, Swiss-Prot and KEGG, and 20,070 (29.06%) were simultaneously annotated by all four databases. Altogether, 45,676 (66.13%) unigenes were successfully annotated in public databases (Fig. 4). The other 23,395 (33.87%) unigenes that were not annotated might be the novel genes or previously not known in these public databases. The E-value distribution of the top hits in the NR database showed that 43% of the annotated unigenes showed significant homology to previously deposited sequences (less than 1.0E-50), and 22% ranged from 1.0E-150 to 0 (Fig. 5A). Of the unigenes with significant homology 41% had identities of greater than 80% and 34% had identities which ranged between 60%–80% (Fig. 5B). The top-hit species distribution of gene annotations showed the highest homology to Zebrafish (*Danio rerio*) with 17,789 unigenes matched, followed by Atlantic salmon (*Salmo salar*) and western clawed frog (*Xenopus tropicalis*) (Fig. 5C). Additionally, 8,292 (86.16%) of the unigenes of more than 1,000 bp in length had BLAST matches, whereas only 3,213 (32.44%) of unigenes shorter than 200 bp did (Fig. 5D), indicating that longer unigenes were more likely to obtain BLAST matches in the protein databases, which was also reported by [35], [43], [44]. The shorter sequences may not be long enough to show sequence matches or may lack a representative protein domain, resulting in false-negative results [44].

By means of performing BLASTX searches against these protein databases, sequence directions and protein coding regions of 35,820 nonredundant unigenes were decided. Furthermore, 1,930 unigenes were predicted the directions and protein coding regions by using ESTScan software [45].

### Functional Classification of Miiuy Croaker Unigenes by GO, COG, and KEGG

**Figure 6:**
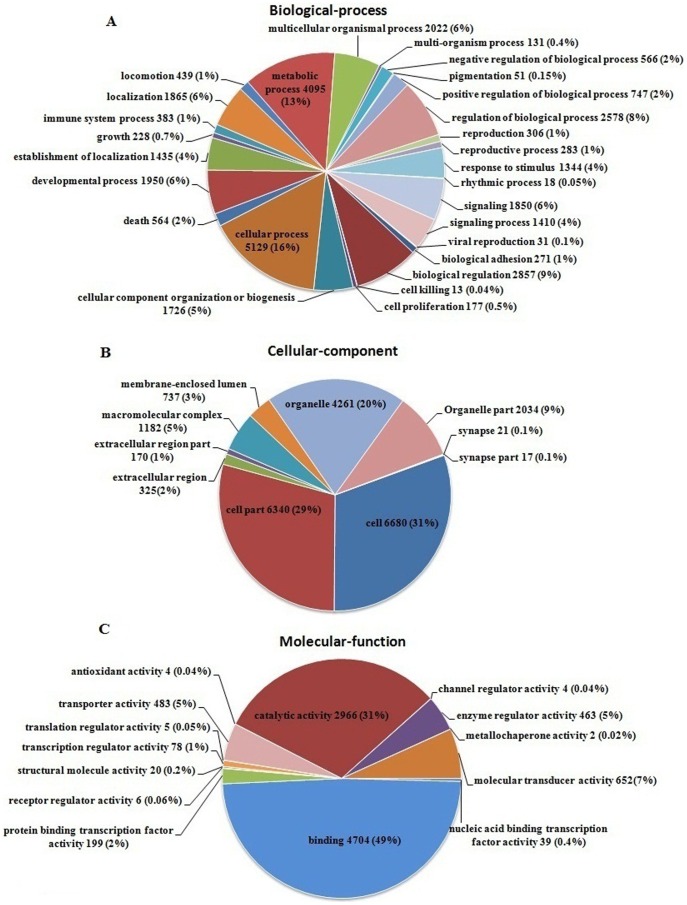
Gene Ontology classifications of assembled unigenes. Unigenes were assigned to three classifications: (**A**) biological processes, (**B**) cellular components and (**C**) molecular functions. In total, 8,423 unigenes with BLAST matches to known proteins were assigned to gene ontology.

Gene Ontology (GO) is an international standardized gene functional classification system which provides a dynamic-updated controlled vocabulary to annotate and analyze the functions of a large number of genes and their products in any organism [46]. Based on NR annotation, the Blast2GO [47] software was used to get the GO annotation, and then a web tool WEGO [48] was used to obtain the GO functional classification for these annotated unigenes. In total, 8,423 of the 35,743 unigenes matched in NR database were classified into 51 function categories under the three ontologies of GO (biological process, cellular component, and molecular function). For each unigene could be assigned to more than one GO terms, altogether 32,469 unigenes were assigned to biological process as the majority, followed by the cellular component (21,767 unigenes) and molecular function (9,625 unigenes). Among the biological process category, cellular process (5,129 unigenes, about 16%) and metabolic process (4,095 unigenes, about 13%) were predominant groups. It was also noteworthy that a large number of genes (383 unigenes) were predicted to be involved in immune system process (Fig. 6A). In the cellular components category, cell (6,680 unigenes, approximately 31%) and cell part (6,340 unigenes, approximately 29%) were prominently represented, followed by organelles (4,261 unigenes, about 20%) and organelles part (2,034 unigenes, about 9%) (Fig. 6B). Under the category of molecular function, 4,704 unigenes (approximately 49%) were assigned to binding, followed by catalytic activity (2,966 unigenes, about 31%), and molecular transducer activity (652 unigenes, about 7%) (Fig. 6C).

**Figure 7:**
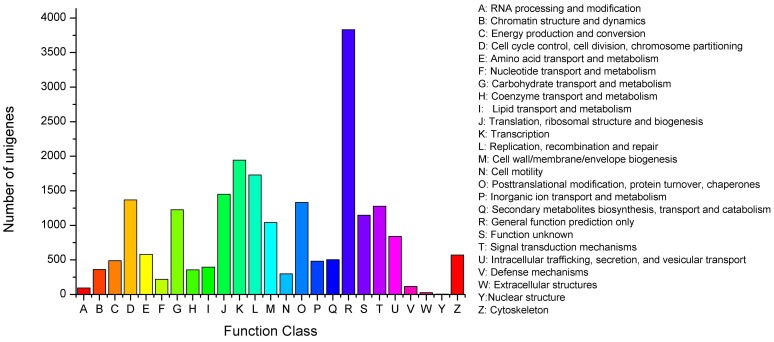
Histogram presentation of clusters of orthologous groups (COG) classification. A total of 21,662 unigenes were assigned the Cluster of Orthologous Groups classification and classified into 25 functional categories (E-value ≤1.0E-5).

All assembled unigenes were further annotated based on the Clusters of Orthologous Groups (COG) database for functional prediction and classification. Altogether, 21,662 unigenes were assigned the Cluster of Orthologous Groups classification, which could be grouped into 25 functional categories (Fig. 7). The cluster for “General function prediction only” (3,830, 17.68%) represented the largest group, followed by “Transcription” (1,942, 8.97%) and “Replication, recombination and repair” (1,724, 7.98%), and transcripts associated with “Translation, ribosomal structure and biogenesis” (1,449, 6.69%), “Cell cycle control, cell division, chromosome partitioning” (1,367, 6.31%) and “Posttranslational modification, protein turnover, chaperones” (1,332, 6.15%) were common, whereas the percentages of three groups, “Nuclear structure”, “Extracellular structures and modification” and “RNA processing and modification” were less than 1.00% (Fig. 7). The category of “Defense mechanisms” (116, 0.98%) might be closely related to miiuy croaker immune defense. The most abundant type of functional description in this category was “ABC-type multidrug transport system” [49], followed by “Na+-driven multidrug efflux pump”.

**Figure 8:**
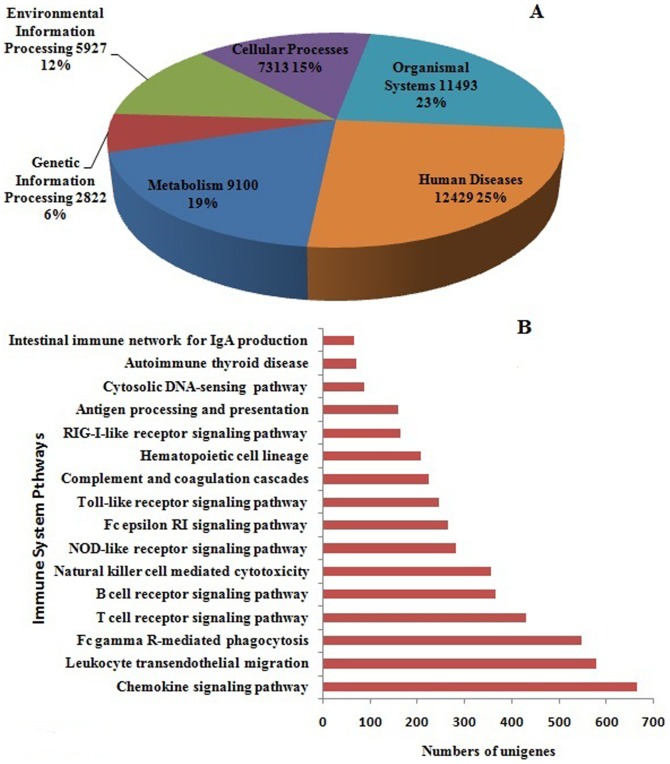
Pathway assignment based on KEGG. (**A**) Distribution of assembled unigenes based on 6 main categories pathways. (**B**) Classification based on Immune System classification.

The Kyoto Encyclopedia of Genes and Genomes (KEGG) is the database that can help to analyze the metabolic pathways of gene products and understand the biological functions and interactions of genes. Based on a comparison against the KEGG database using BLASTX with an E-value cutoff of <10^−5^, a total of 23,927 unigenes with significant matches in the database were assigned to 123 KEGG pathways (Table S1). These pathways were distributed to 6 main categories: Metabolism (9,100 unigenes, 19%), Genetic Information Processing (2,822 unigenes, 6%), Environmental Information Processing (5,927 unigenes, 13%), Cellular Processes (7,313unigenes, 15%), Organismal Systems (11,493 unigenes, 23%) and Human Diseases (12,429 unigenes, 25%) (Fig. 8A). In addition, according to the Immune System classification, 4,719 unigenes were classified into 16 pathways, which include Chemokine signaling pathway, Leukocyte transendothelial migration, T cell receptor signaling pathway and Toll-like receptor signaling pathway. The Immune System pathway with the largest number of unigenes mapped to it was “Chemokine signaling pathway” (Fig. 8B). These pathways indicated the active immune processes and provided a valuable resource of immune transcripts in miiuy croaker.

### Identification of Immune-relevant Genes

It was widely believed that innate and adaptive immunity in teleosts was established about 470 million years ago [50], [51]. However, the immune system study of miiuy croaker is still in its infancy due to lack of transcriptomic and genomic resources. To devote to better understanding the immune system in miiuy croaker, we found 498 unigene sequences showing significant homology to immune-relevant genes based on the annotations of NR database. Moreover, unigenes in the GO categories “response to stimulus”, “immune system process” and KEGG classifications “immune system”, “immune diseases” were also used to identify these immune-relevant genes. We divided these genes into fourteen categories on the basis of the putative function, such as “cytokines and cytokine receptors” (128), “immunoglobulin and receptors” (66), “cell apoptosis and cell cycle” (54) and “transcription factors for immune response” (37). The detailed classification, putative function and matched species of these immune-related genes were given in Table S2. Except for some representative immune genes, such as chemokines [9]–[11], C9 [12], MHC I, II [13], [14], cathepsins [17], [18] etc, most of these immune-related genes identified in this report were not studied in miiuy croaker. The 193 immune-related genes identified in previous study [18] were far less than the 498 immune-related genes obtained from this study. For instance, some immune genes like complement component C4, CC chemokine CK5, CC chemokine CK8, cathepsin B. F. H. S. Z, C-type lectin 2 and 7, caspase genes, novel immune-type receptor 6, 9, 12, 13, et al. that previously not known in miiuy croaker were acquired. These immune-related genes and immune mechanisms will be further studied in-depth in our laboratory.

### Development and Characterization of cSSRs and SNPs in the Miiuy Croaker Transcriptome

**Figure 9:**
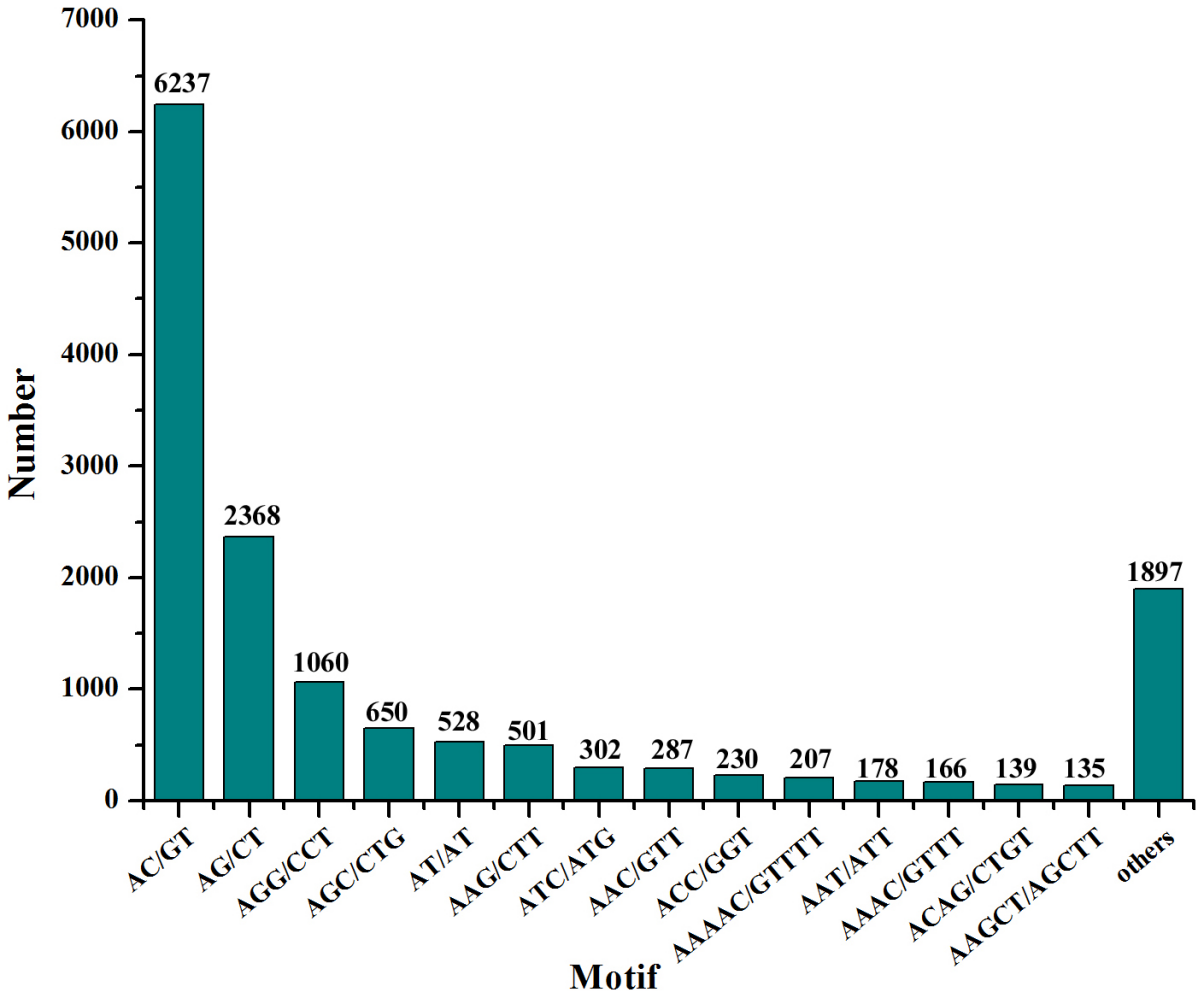
Frequency distribution of cSSRs based on motif sequence types. The most abundant repeat motif in our cSSRs was AC/GT (41.90%), followed by AG/CT (15.91%), AGG/CCT (7.12%) and AGC/CTG (4.37%) among 284 motif sequence types.

From the 69,071 unigenes, a total of 11,251 unigenes containing 14,885 cSSRs were identified with 2,697 of the sequences containing more than one cSSR and 2,179 cSSRs were categorized as compound repeats by using the MISA Perl script (Table 3). Mononucleotide repeats or cSSR loci with length less than 10 bp were not included in this study. Furthermore, the frequency distribution of these putative cSSRs was about every 2.7 kb of miiuy croaker unigene sequence. Of these cSSRs, the most abundant type of repeat motif was Di-nucleotide repeats (9,145), followed by Tri- (3,317), Penta- (1,043), Tetra- (784), and Hexa-nucleotide (596) repeat units (Table 3). The frequencies of cSSRs with different numbers of tandem repeats were collected and are shown in Table 4. The cSSRs with five tandem reiterations (5,476) were the most abundant, followed by six tandem repeats (2,466) and seven tandem repeats (1,350). Within the searched cSSRs, we identified 284 motif sequence types, Di-, Tri-, Tetra-, Penta- and Hexa-nucleotide repeats were 4, 10, 30, 90 and 150 types, respectively (Table S3). The most dominant repeat motif in these cSSRs was AC/GT (41.90%), followed by AG/CT (15.91%), AGG/CCT (7.12%) and AGC/CTG (4.37%). While, very few CG/CG (12 0.08%) repeats were identified in our database (Fig. 9).

**Table 3:** Summary of cSSR searching results.

Searching item	Numbers
Total number of sequences examined	69,071
Total size of examined sequences (bp)	39,541,954
Total number of identified SSRs	14,885
Number of sequences containing more than 1 SSR	2,697
Number of SSRs present in compound formation	2,179
Di-nucleotide	9,145
Tri-nucleotide	3,317
Tetra-nucleotide	784
Penta-nucleotide	1,043
Hexa-nucleotide	596

**Table 4:** Length distribution of cSSRs based on the number of repeat units.

Repeat numbers	Motif length					Total
	Di	Tri	Tetra	Penta	Hexa	
3				840	488	1,328
4			446	138	75	659
5	3,702	1,579	146	29	20	5,476
6	1,609	761	70	15	11	2,466
7	896	411	37	4	2	1,350
8	665	214	25	11		915
9	458	131	15			604
10	356	76	38			470
11	261	44	1	3		39
12	228	37		2		267
13	174	42	3	1		220
14	150	4	1			155
15	112	5				117
16	74	6				80
17	95	1				96
18	172	1	2			175
19	85	1				86
≥20	108	4				112
Total	9,145	3,317	784	1,043	596	14,615

Eighty-seven primer pairs were designed and synthesized from the randomly selected unique sequences for further assessing the quality of the putative SSRs generated in our study and development of new microsatellite markers. Among, 58 primers successfully amplified PCR products at the expected sizes, with forty-one produced a single product, while the other seventeen primer pairs amplified multiple products. Twenty-five of the 58 microsatellite loci were examined showing allelic polymorphism across 10 wild miiuy croaker individuals. Then we assessed the molecular characterization of the polymorphic loci among 30 wild individuals of miiuy croaker. Allele number of these loci ranged from 2 to 9 with an average of 3.88 (Table 5). The observed heterozygosity (*Ho*) ranged from 0.100 to 1.00 with an average of 0.433, while the expected heterozygosity (*He*) ranged from 0.095 to 0.784 on average of 0.489. Polymorphism information content (*PIC*) values of per locus varied from 0.005 to 0.991 with an average of 0.49 (Table 5). Although the alleles of the novel developed loci was a little lower than genomic-SSR markers of which the alleles were respectively tested with an average number of 5.12 and 5.67 in the previous studies [19], [20].

**Table 5:** Characterization of 25 polymorphic cSSRs loci in miiuy croaker.

Locus	Primer sequence	Repeat type	Tm(°C)	Size range/bp	No.of alles	*Ho/He*	*PIC*
							
Unigene25	F:TCTTCGGGCTCACTTCTC	(GGTC)4(TG)5	50	196–204	3	0.429	0.355
	R:CTTGGTCTTTGATTTTGC					0.582	
Unigene30	F:TGTCAATCATAAGCCTCG	(AAAT)5	50	188–196	3	0.353	0.116
	R:TTCAGCCTGTACCTGTGC					0.305	
Unigene56	F:AGGGAAAGGGTTGGGATA	(GAA)5	52	156–168	4	0.333	0.486
	R:GTTGGTGCTGTTGCGAAT					0.542	
Unigene64	F:CTTAGCCAGCAAGTGAAT	(CTTC)18	50	208–298	9	0.778	0.717
	R:ATAACACCGTGACCCATA					0.836	
Unigene129	F:GTCGCAGCCGTGAGGATA	(GTG)7	52	175–181	4	0.2	0.265
	R:TCAGAACGGAGCCAGTCA					0.27	
Unigene188	F:GAACGAAAGCAGCGAACA	(AC)8	52	222–242	3	0.643	0.501
	R:TCCACAGCCAGTCCAGAG					0.589	
Unigene192	F:CTCCTCGTTTGGATTGTT	(TG)12	52	276–298	6	0.563	0.966
	R:TCGGTAAACTTCCCTCTA					0.727	
Unigene199	F:TCGTCCTATCTGCTGTGG	(TG)7	52	117–123	2	0.308	0.226
	R:ACCTTGCAGGATGTGAGT					0.26	
Unigene259	F:GCGACTCAACATCAACCA	(GT)8	52	115–127	3	0.8	0.363
	R:CTTCCTTCCCTCCTTTCC					0.598	
Unigene298	F:ATTGTTGGTTTTGGAGGC	(TG)7	52	230–246	6	0.556	0.743
	R:ATGTATGCTCGCTGCTTT					0.661	
Unigene340	F:AAGCATCTGAATCCTCTGT	(TG)9	52	180–190	4	0.389	0.921
	R:TCCTCTACCCGAACCTCT					0.511	
Unigene387	F:GTCCCGAGTACGGATGAA	(CA)12	52	230–246	2	0.167	0.207
	R:AGGTTCCCTGCCCTACAT					0.239	
Unigene929	F:CTCTGACTCCACAGTTCACAC	(CA)7	56	184–190	2	0.1	0.09
	R:GGTGCACTATGTGCTTTAAGT					0.095	
Unigene988	F:TCCAACTTTATCCACTGTGTC	(CTG)6	53	153–162	3	0.444	0.366
	R:CTCTTCATGGCGACATAACT					0.44	
Unigene1043	F:CCCAAGAAGTGACATGATTT	(TG)8	51	158–162	3	0.154	0.379
	R:CTTTGTTGTGAAGGTCTGAAC					0.447	
Unigene1048	F:TACATCACATGAGGTGAACG	(GAT)6	53	146–152	3	0.111	0.053
	R:ACCTCCAGTGCAGTTATACAG					0.107	
Unigene1145	F:TAAGTGTATCGACCAGGTGAG	(CA)7	55	149–153	2	0.385	0.394
	R:ACTCATTCATTCAGGTCACAG					0.453	
Unigene1196	F:CTCTCATTCTGTGCTCGTCT	(GT)7	55	149–155	5	0.643	0.932
	R:GGTGCAGTAGCTTAATCACAC					0.589	
Unigene1231	F:AGATGGAGATGCGTACTTGT	(CA)14	55	142–158	6	0.667	0.991
	R:CCTGTGCAGACAGTAGGTTAC					0.711	
Unigene1256	F:GTGTGTGGTTTCACCATTTA	(GT)7	55	177–179	2	0.1	0.005
	R:GCGCTTACTCACTCATACACT					0.095	
Unigene1269	F:CCTAATCTGTCGGGATAAACT	(AC)10	55	171–191	6	1	0.957
	R:CTTTGCTCAACTCACTGATTC					0.784	
Unigene1361	F:CCAAGCTCTCTTTATCTTCCT	(TC)7	55	169–173	3	0.105	0.571
	R:CCTCCATGTTCATCAGAGTT					0.575	
Unigene1404	F:CATGTCTGAGGCACAATGTAT	(TTA)7	56	130–140	4	0.412	0.517
	R:GTCACAGCTTGTTTTGGTACT					0.659	
Unigene1461	F:CTCACTGAAACACCAACAATAG	(AG)10	56	149–163	6	0.706	0.73
	R:CCCCTCTCTCTCTCTTTCTCT					0.701	
Unigene1496	F:AATGGCAGTCTGACAGATAAG	(CA)7	56	125–133	3	0.471	0.384
	R:CCGACTGCTCTTCTTCTATTA					0.443	
Average					3.88	0.433	0.49
						0.489	

**Figure 10:**
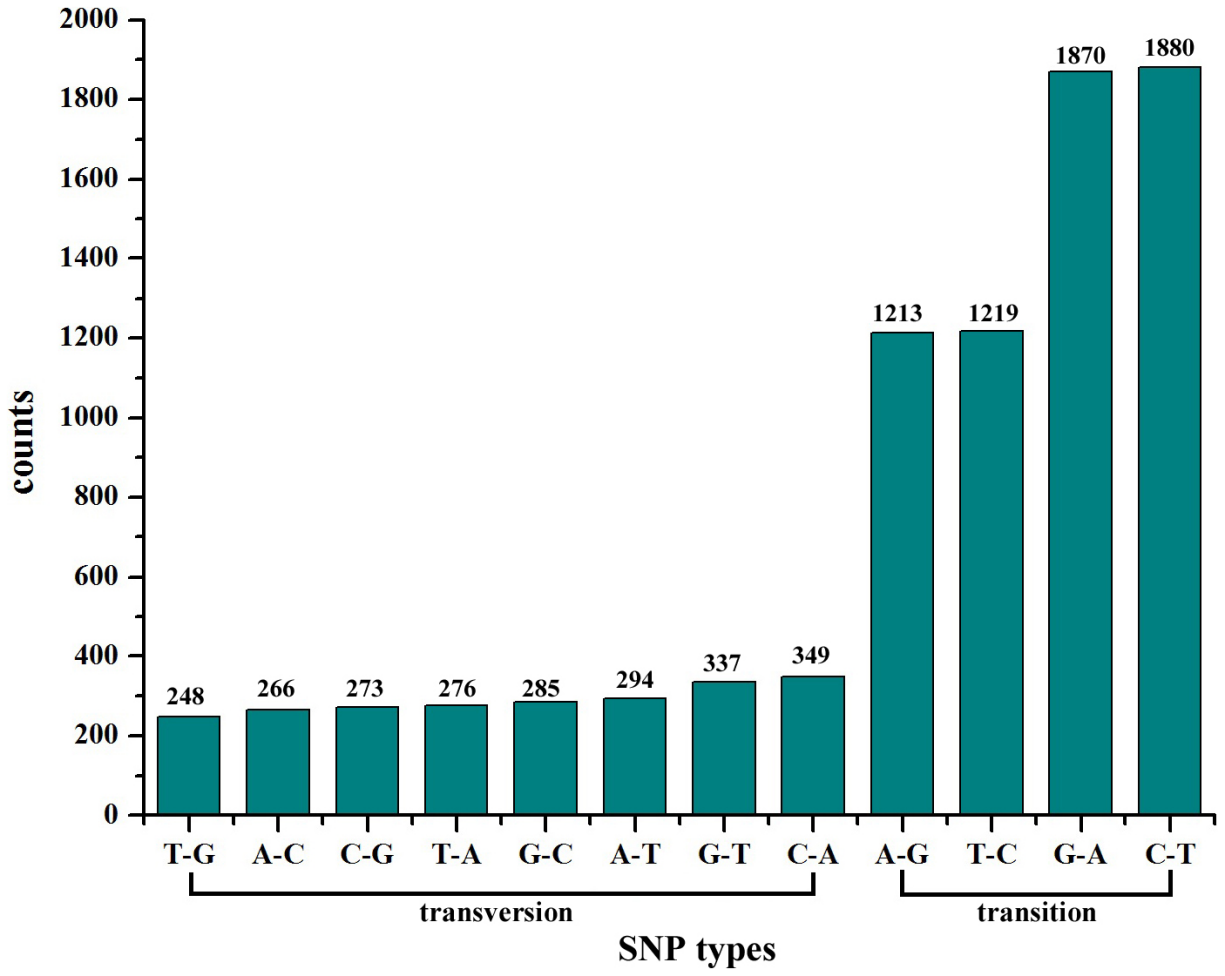
Frequency distribution of SNPs based on different types. A total of 8,510 putative single nucleotide polymorphisms (SNPs) included 2,328 transversions and 6,182 transitions.

Additionally, we also identified a total of 8,510 predicted single nucleotide polymorphisms (SNPs) by mapping against 69,071 reference unigenes, including 6,182 transitions and 2,328 transversions. The number of different transition types (A/G, C/T) was similar, and also a similar number of the four transversion types (A/T, A/C, G/T, C/G) were found (Fig. 10). These SNPs should be very useful for further genetic or genomic studies and marker development on this species [36]. While, the false positives and sequencing errors of all the potential SNP molecular markers should be to eliminate by validation. The cSSR and SNPs molecular markers we identified in miiuy croaker by deep transcriptome sequencing using next generation sequencing will provide a wealth of data for further genetic study as well as mapping and tagging in genes and molecular assistant breeding.

## Conclusion

To our understanding, this is the first time using Illumina paired-end sequencing technology for miiuy croaker transcriptome *de novo* sequencing and assembly without reference genome. Except for characterizing the transcriptome of miiuy croaker, we obtained abundant resources for new gene discovery and molecular markers development for further study. In particular, those immune-relevant genes could provide significant resources to understand the immune systems and prevent disease of miiuy croaker. The putative molecular markers identified in this study can be used for constructing genetic linkage maps and researching gene-based association in miiuy croaker. Besides, our study confirmed one more time that Illumina paired-end sequencing is a fast and cost-efficient tool to discover novel genes and develop molecular markers in non-model organisms, especially those with vast and complicated genomes.

## Materials and Methods

### Ethics statement

This study has been approved by the permission from the Animal Welfare Committee.

### Biological Materials and RNA Extraction

Healthy miiuy croakers were obtained from Zhoushan Fisheries Research Institute (Zhejiang, China). The samples were removed from three tissues (liver, spleen and kidney) of five individuals, and then immediately frozen in liquid nitrogen and kept at −80°C until use. TRIzol based RNA isolation protocol was used to extract RNA from each tissue according to the manufacturer's instructions. RNA concentration was quantified using a SmartSpec Plus spectrophotometer, and potential degradation was examined by 1% agarose gel electrophoresis. Equimolar concentrations of extracted RNA from different samples of miiuy croakers were mixed to create an at least 20 μg RNA pool for cDNA library construction.

### cDNA Library Construction and Sequencing

Ahead of cDNA library construction, the total RNA was treated with DNase I, and magnetic beads with Oligo (dT) were used to enrich poly (A) mRNA from it. For fear of priming bias when synthesizing cDNA, the purified mRNA was disrupted into short fragments by adding to fragmentation buffer. The first-strand cDNA was synthesized by using the mRNA fragments as templates, and random hexamers as primers, then buffer, dNTPs, RNaseH, and DNA polymerase I were used to synthesize the second-strand cDNA. Subsequently, the synthesized double-stranded cDNA was subjected to end-repair, add poly (A) and connect with sequencing adapters after purifing with QIAquick PCR extraction kit and eluting with EB buffer. Finally, the suitable fragments purified by agarose gel electrophoresis were selected as templates for PCR amplification. An Illumina HiSeq™ 2000 sequencing system was used to sequence the organized cDNA library at the Beijing Genome Institute.

### Analysis of Illumina Sequencing Results

The sequencing-obtained original image data was converted into raw reads by means of base calling. Among them, the reads with adaptor, repeated reads and low-quality reads (with more than 50% Q≤20 bases) which may affect the assembly and analysis were firstly removed. These remaining high-quality reads were used to assemble the transcriptome of miiuy croaker with SOAPdenovo [41]. Ultimately, unigenes without redundancy, containing the least Ns and not being extended on either end were obtained. The sequence data were submitted to NCBI Sequence Read Archive under the accession number of SRA122355. And the assembled sequences have been deposited in the NCBI transcriptome shotgun assembly (TSA) database. This transcriptome shotgun assembly project has been deposited at DDBJ/EMBL/GenBank under the accession GARA00000000.

Unigenes were searched against the NCBI nonredundant, Swiss-Prot, and KEGG protein databases and nonredundant nucleotide databases using BLASTX and BLASTN algorithm with an E-value threshold of 1.0E-5 to determine the sequence directions and protein coding regions. Based on NR annotation, the Blast2GO [47] software was used to get the GO annotation, and then a web tool WEGO [48] was used to obtain the GO functional classification for these annotated unigenes. The unigenes were further annotated based on the COG database for functional prediction and classification, and aligned to KEGG database for pathway assignments.

### RT-PCR amplification of transcripts

To validate the assembly of the transcriptome and the presence of novel transcripts detected by Illumina paired-end sequencing in miiuy croaker, twelve selected sequences were used for expression analysis by RT-PCR. The twelve sequences were randomly selected from different length regions of the annotated unigenes. Total RNA was prepared from the tissues (liver, spleen and kidney) of adult individuals using Trizol reagent in accordance with the manufacturer's instructions. cDNA was synthesized utilizing a QuantScript RT Kit according to the manufacturer's protocol, and then was stored at −20°C for later. Specific primer pairs for cDNA amplification were designed by BatchPrimer3 [52] according to the transcript sequences. Each RT-PCR was performed in a 25 μl reaction volume consisting of 1 μl cDNA, 2.5 μl 10× reaction buffer, 2 μl dNTPs (2.5 mM), 1 μl of each primer (10 μM), 0.2 μl *Taq* polymerase (5 U/μl) and 17.3 μl dd H2O. The amplification conditions were varied according to the different annealing temperature and time and extension time for different primer pairs and the different amplification length. The PCR products were determined by 1% agarose gel electrophoresis using DNA markers.

### Identification of cSSR and SNP

SSR motifs both perfect and compound were identified using MIcroSAtellite (MISA, http://pgrc.ipk-gatersleben.de/misa/) [53]. We searched for Di-, Tri-, Tetra-, Penta- and Hexa- nucleotides repeats with a minimum of 5, 5, 4, 3, and 3 repeats, respectively. Putative single nucleotide polymorphisms (SNPs) detection was performed using SOAPsnp [54] software by mapping against 69,071 reference unigenes.

### Primer Validation and Polymorphism Assessment

Primer pairs flanking the SSR motifs were designed using BatchPrimer3 [52] and synthesized by the company Genscript. Primer validation was carried on genomic DNA of miiuy croaker by PCR reactions. Each PCR reaction consisted of 1 μl of 10× reaction buffer, 0.8 μl dNTPs, 0.6 μl of the forward and reverse primers, 1 μl template genomic DNA and 0.1 μl of *Taq* polymerase (5 U/μl) in a finally 10 μl reaction mixture. And the PCR amplification conditions: denaturation at 95°C for 5 min, followed by 30 cycles of 95°C for 30 sec, 55°C for 30 sec, and extension for 30 sec at 72°C, finally followed with a final extension for 5 min at 72°C, and then holding at 4°C. Ten individuals were used to assess the polymorphism primers and then perfect amplified loci were examined the genetic characterization by thirty individuals. Allele number of these loci and mean allele number were calculated using Popgene version 1.32 [55], and the data *PIC* was analyzed using PIC_CALC and GenAlex6 [56].

## Supporting Information

Table S1 **KEGG categories of 23,927 nonredundant unigenes in miiuy croaker.**
(XLS)

Table S2 **The detailed classification, putative function and matched species of these identified immune-relevant genes.**
(XLS)

Table S3 **Identified cSSRs in miiuy croaker.**
(XLS)
